# Perceptions of Stress, Well-Being, and Intervention Preferences Among Parents Affected by Major Stressors

**DOI:** 10.3390/healthcare13182366

**Published:** 2025-09-20

**Authors:** Nada M. Goodrum, Julie K. Nguyen, Diamonde McCollum, E. Rebekah Siceloff, Brianna Tennie, Sara delMas, Ronald J. Prinz

**Affiliations:** 1 Department of Psychology, University of South Carolina, Barnwell College, 1512 Pendleton Street, Suite #220, Columbia, SC 29208, USA; 2 Research Center for Child Well-Being, University of South Carolina, Columbia, SC 29208, USAbtennie@mailbox.sc.edu (B.T.); 3 National Crime Victims Research and Treatment Center, Medical University of South Carolina, Charleston, SC 29403, USA

**Keywords:** parenting, stress, prevention, intervention, mixed methods, child well-being

## Abstract

**Background/Objectives**: Children’s social–emotional difficulties and unhealthy lifestyle behaviors co-occur but are rarely addressed concurrently in parent-based interventions. These problems are exacerbated by family stressors (e.g., parental trauma, mental health, substance misuse, illness, financial strain, racism), which further compound existing health and healthcare inequities for families experiencing marginalization who are more likely to face major stressors. Yet, most parent-based interventions do not sufficiently address parents’ own stress and self-regulation. To inform prevention efforts to address these gaps, this mixed methods formative needs assessment explored strengths, challenges, and intervention preferences of parents dealing with major stressors, informing parent-based prevention to improve child social–emotional and physical health. **Method**: A sociodemographically diverse sample of parents/caregivers (N = 46) who had a child aged 3–9 years and ≥ two major stressors completed surveys assessing child well-being, parenting, parental stress, self-regulation, and intervention preferences. A subsample (n = 24) completed qualitative interviews further exploring these areas. **Results**: Parents expressed high interest in programming on emotion regulation, mindfulness, dealing with trauma, and reducing stress while strengthening children’s social–emotional and physical health. **Conclusions**: Results underscore the need to address caregivers’ own emotion-related skills to promote children’s well-being. Findings inform implementation and evaluation of a preventive program to improve child health, promote positive parenting, and address parental stress through self-regulation and coping. By aligning with community needs and preferences, parenting interventions that simultaneously address parental well-being and stress may be a promising avenue for improving equitable access to and quality of healthcare for families experiencing marginalization and stress.

## 1. Introduction

Despite some successful prevention efforts, social–emotional difficulties and unhealthy lifestyle behaviors persist among children in the United States. These challenges tend to co-occur and exacerbate one another in childhood [1] and are especially pronounced among families dealing with major stressors [2,3]. The prevalence and clustering of unhealthy lifestyle behaviors and social–emotional problems increase with age [4], suggesting the need for prevention efforts to disrupt this trajectory early in childhood. Several family-based preventive interventions can effectively improve parenting skills, enhance parent–child relationships, and in turn promote children’s social–emotional health [5] and, to a lesser extent, children’s lifestyle behavior health, though evidence in this domain is more limited [6]. However, families dealing with stressors face barriers to benefitting from these interventions [7], in part because they may not sufficiently address family stress and effective coping. Further, these interventions typically target child outcomes in either social–emotional or healthy lifestyle domains, but not both concurrently. The current mixed methods study explored (a) the impact of major stressors on families and resilience factors and (b) parents’ preferences and opinions regarding parenting support programs to jointly address child social–emotional and lifestyle health.

### 1.1. Prevalence and Overlap Between Social–Emotional and Lifestyle Behavior Problems

Social–emotional functioning is a broad construct that includes children’s skills in emotion regulation, behavioral regulation, and peer and social interactions [8]. Difficulties in this domain often underlie mental, emotional, and behavioral disorders. An estimated 16.5% of U.S. children (nearly 8 million) have a mental health disorder [9]. Unhealthy lifestyle behaviors in children can include sedentary behavior, excessive screen time, low physical activity, poor nutrition, and sleep problems. Two-thirds (66.3%) of children aged 2–5 years and 83.3% of children aged 6–11 years engage in at least one of these unhealthy lifestyle behaviors, and 30–50% exhibit two or more [4]. Additionally, nearly half (41.9%) of U.S. children experience sleep problems, which are correlated with other aspects of physical and behavioral health [10]. Social–emotional difficulties and unhealthy lifestyle behaviors both predict adverse short- and long-term consequences. Social–emotional problems in childhood may lead to mental health disorders, substance use, sexual risk behavior, HIV and other STIs, academic problems, suicidality, and physical health problems in adolescence and adulthood [11]. Unhealthy lifestyle behaviors increase later risk for cardiovascular disease, obesity, cancer, diabetes, mood disorders, inattention, hyperactivity, low self-esteem, and academic problems [1].

### 1.2. Child Well-Being Among Families Dealing with Major Stressors

In families facing major stressors such as parental trauma history, mental health difficulties, substance misuse, HIV, frequent interpersonal racial discrimination, and economic strain, children are more likely to exhibit emotional, behavioral, social, and physical health problems [12,13]. These concerns are often partially due to broader socio-structural factors, such as racism embedded in societal systems and structures, intergenerational poverty, and thwarted access to quality healthcare and other resources. At the individual family level, parenting is one pathway by which stressors negatively impact child health. Belsky’s [14] process model of parenting posits that parenting quality is determined in part by parents’ own psychological resources as well as contextual sources of stress and support. Research indicating negative effects of parental stressors on parenting behavior supports this theory. Relatedly, the family stress model describes processes by which economic strain negatively impacts children indirectly through parents’ distress and disrupted parenting [3]. Consistent with both frameworks, major stressors make it more challenging for parents to engage in effective parenting. Further, parents impacted by stressors are less likely to be able to buffer their children against adversity. Importantly, these challenges disproportionately affect Black, Indigenous, and People of Color (BIPOC) families, with structural racism perpetuating adversity in these communities [15,16]. Structural racism and other forms of oppression also create barriers to accessing high-quality healthcare services, including both parenting services and mental health services [15]. By providing healthcare services that integrate positive parenting skills with parental mental health and well-being, parenting interventions that address parent stress among BIPOC families may be a path to mitigating child health inequities.

**Figure 1:**
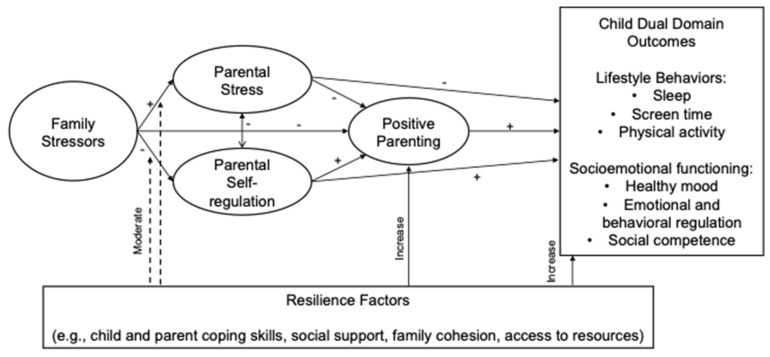
Conceptual model informed by the family stress model, process model of parenting, and resilience theory.

The current study focused on families affected by six parental stressors that have been found to pose challenges for positive parenting, child health outcomes, and engagement in preventive interventions. These stressors include parental (1) history of trauma exposure, (2) mental health difficulties, (3) substance misuse, (4) HIV, (5) racial discrimination, and (6) economic strain. These stressors were selected because they frequently co-occur, exacerbate one another, and have been conceptualized as a syndemic [17]. Although there are many other stressors that can affect children and families, these six were chosen because of their syndemic nature and salience for health equity. Though there is substantial evidence that each of these major stressors negatively impacts families [18,19,20], these stressors are rarely examined within the same study despite high rates of co-occurrence and potentially cumulative effects. Further, studies examining each of these stressors often adopt a deficit approach while neglecting to examine resilience factors that may buffer children and families against the negative effects of these stressors. Resilience refers to a dynamic process promoting positive adaptation in the context of adversity [21] and can include factors at the individual child, parent, family, or community levels. Figure 1 displays a hypothesized conceptual model that, though not tested in this exploratory study, outlines the theoretical framework guiding this line of work.

### 1.3. Parental Self-Regulation and Stress

Parental self-regulation (SR) is a key process underlying parents’ coping and positive parenting. Parental SR refers to parents’ ability to monitor their own and their child’s thoughts, feelings, and behaviors to engage in goal-directed parenting behavior suitable for the demands of the current context [22]. It includes skills such as recognizing the child’s needs, deciding how to respond to problem behavior, choosing an appropriate tone of voice and body language, remaining aware of the immediate context and situation, and feeling confident about one’s parenting abilities. Parental SR underlies positive parenting and is thus related to children’s healthy development [23].

Parental SR may facilitate maintenance of gains in parenting preventive interventions because parents must take ownership of applying the parenting skills they learned [22]. Yet, it is often treated as an implicit mechanism of change rather than an explicit intervention target. In contrast to parenting programs that are directive with an emphasis on teaching knowledge, a parenting program that seeks to directly target SR would emphasize parents’ autonomy and problem-solving skills in selecting and flexibly applying evidence-based parenting strategies. This approach may be particularly suitable for parents affected by major stressors who may have difficulty developing or applying parental SR strategies, given the competing demands and cognitive load of stress. In addition to supporting parenting skills gains, an SR approach may increase intervention engagement by equipping parents with skills to problem solve barriers to treatment attendance (e.g., scheduling, child care, transportation) and by empowering parents to apply positive parenting practices in a manner aligned with their culture and values.

### 1.4. Successes and Challenges of Existing Parent-Based Prevention Programs

Parent-based preventive interventions can be effective in improving parenting quality, with some programs finding effects lasting 15 years after program completion [5]. Parenting programs have been shown to improve parenting practices, including appropriate discipline, communication, relationship quality, involvement, monitoring, and reduced conflict and coercive behavior. Several parenting programs also show evidence of improving child behavior across domains, including reducing externalizing and internalizing problems and promoting child self-regulation, physical health outcomes, and academic success.

Unfortunately, however, most parenting support programs do not directly address parent/family stress or parental self-regulation, which may diminish the benefits of these programs for families affected by major stressors. Some preventive interventions show promise in targeting parenting among families facing adversity, with several demonstrating efficacy in improving child, parent, and/or family outcomes [24,25,26]. However, many parent-based preventive interventions are limited in (1) not directly addressing parents’ own stress and coping and (2) not jointly targeting both social–emotional and physical health outcomes in children. Filling these critical gaps might improve intervention engagement among families who typically face multiple barriers when accessing and completing parenting programs. A key goal is to enhance preventive intervention impact by integrating (a) parental self-regulation and stress reduction and (b) children’s lifestyle behavior health and social–emotional functioning in the context of positive parenting skills.

### 1.5. Parents’ Preferences and Opinions Regarding Parenting Support Programs

Some research has indicated that parental engagement within prevention or intervention programs can be increased by aligning content and delivery with parents’ preferences [27,28]. In a sample of parents of preschool-aged children, parents indicated a preference for programs that include skill-building related to child and parent outcomes, including children’s behavioral and social skills, positive parenting, and other parent skills [28]. Parents’ beliefs regarding the most effectual delivery methods (e.g., individually tailored programs, home visits) vary based on parents’ race/ethnicity, gender, perceived level of distress, family size, thoughts about their children, and parenting style, among other factors [27,28]. For example, parents from ethnic minority backgrounds were found to be more likely than their White counterparts to value programs that include skill development for parents beyond parenting, such as educational attainment and job skills [28]. Parents with more concerns about their children’s emotional and/or behavioral difficulties may be more willing to seek support and might prefer face-to-face formats [27], though other data show strong acceptability and engagement with virtual delivery [29]. The implication is that the success of parenting support programs can be bolstered by addressing both parent and child skills, as well as by considering parents’ modality preferences. Accordingly, this study gathered mixed methods data on intervention preferences to inform delivery of a parent-based preventive intervention within a local community of families affected by adversity.

### 1.6. Current Study

Informed by the process model of parenting [14], the family stress model [3], and resilience theory (Masten, 2021), the current study focused on child and parent well-being in the context of adversity to inform future prevention efforts. Given the co-occurrence between child social–emotional and lifestyle behavior difficulties, and evidence that these difficulties may be worsened by parental stressors, one potential avenue for enhancing engagement and outcomes of preventive interventions is directly addressing parental stress in the context of evidence-based parenting support strategies. As a first step toward this goal, the purpose of this mixed methods study was twofold: first, to explore parents’ perceptions of their child’s well-being, the impact of major stressors on their family, and factors that promote resilience and, second, to assess parents’ preferences and opinions regarding parenting support programs to jointly address child social–emotional and lifestyle health. This study was designed to inform a subsequent pilot randomized trial of a parent-based preventive intervention to enhance children’s social–emotional and lifestyle behavior health through promoting positive parenting and parental self-regulation. Given these goals, this study was conducted among a relatively small sample as an exploratory community needs assessment.

## 2. Method

### 2.1. Participants and Recruitment

Participants residing in rural and semi-urban communities in the Southeast United States were recruited through community organizations such as daycares, pediatric and other medical offices, libraries, domestic violence organizations, word of mouth, and online through various social media platforms. Flyers were distributed through these various community organizations in waiting areas and posted on social media pages of community partners. Participants were also referred directly by staff of community agencies. Participant eligibility required: (1) parent or caregiver to a child aged 3 to 9 years old; (2) parent has one or more concerns about the child’s behavior, mood, or lifestyle health; (3) parent is experiencing two or more stressors (i.e., living with HIV, history of traumatic event exposure, mental health difficulties, racial discrimination, substance misuse, financial strain); and (4) English speaking. Eligibility criteria for each stressor are described below. “Parents” refers to all parents and other caregivers in the sample. Among the 57 eligible parents, the most commonly endorsed stressors were trauma exposure (82.5%), mental health (82.5%), financial strain (80.7%), and racial discrimination (22.9%). The 46 caregivers who completed the main study survey, all women, included 45 mothers and 1 grandmother. Eleven eligible participants did not complete the survey and, therefore, were not invited for a qualitative interview. A subsample of 24 caregivers participated in qualitative interviews. Demographic characteristics are summarized in Table 1. The sample was diverse in terms of race/ethnicity and socioeconomic indicators, with over 50% BIPOC families and over 50% reporting less than USD 2000 in monthly income. Participants’ prior participation in parenting programs was not assessed.

### 2.2. Procedures

All study procedures were approved by the university’s Institutional Review Board (IRB). Interested parents were directed to a Qualtrics page that included detailed information about study procedures, an informed consent form, and a screener to assess eligibility. Study staff verified eligibility and texted or emailed the participant a link to complete the 20 min online survey. After the survey was completed, participants were invited to participate in a 30 min phone or Zoom interview. Interviews were conducted with study staff and were audio recorded with participant consent. Parents received USD 20 e-gift cards for completing the survey and interview for a total of USD 40. This compensation amount was approved by the IRB and is not considered coercive for research participation.

### 2.3. Measures

#### 2.3.1. Eligibility Screening Measures

Six measures assessed the presence or absence of caregiver stressors. Sample items for all measures, as well as wording for screening items, are found in the Supplementary Materials (Tables S1 and S2).

**Parent mental health.** Caregivers completed the Patient Health Questionnaire (PHQ-4), a 4-item screening measure of anxiety and depression [30]. Items were measured on a 4-point Likert-type scale ranging from 0 = *Not at all* to 3 = *Nearly every day*. Participants are asked to report on how often over the past two weeks they have been bothered by core anxiety and depression symptoms. Items were summed with higher scores indicating more mood disorder symptoms. Parents met criteria for this stressor if they scored above a 3.

**Trauma exposure.** Trauma exposure was assessed using one item from the Primary Care Posttraumatic Stress Disorder (PC-PTSD-5) [31]. Participants responded “yes” or “no” to a question assessing whether they have ever experienced something unusually frightening, horrible, or traumatic. Participants met criteria for this stressor if they responded “yes”.

**HIV status.** HIV status was assessed with the following question: “Have you been diagnosed with any of the following health conditions? Please select all that apply.” Some responses included “HIV or AIDS”, “cancer”, and “heart disease”. This stressor was met if participants selected “HIV or AIDS.”

**Racial discrimination.** The Everyday Discrimination Scale [32] was used to measure perceptions of racism, with a 5-item measure with a 6-point Likert scale from 0 = *Never* to 5 = *Almost Everyday*. Participants are asked to respond to how often in daily life they experience different discriminatory acts because of their race/ethnicity. Items are summed, with higher scores indicating more experiences of racial discrimination. Participants met criteria for the racial discrimination stressor if they scored 10 or more on the items.

**Alcohol use.** The Alcohol Use Disorder Identification Test-Concise (AUDIT-C) [33] was used to assess alcohol misuse. The 3-item measure asks about quantity and frequency of alcohol consumption and heavy episodic drinking. Each response choice is valued from 0 to 4 and summed for a total scale of 0 to 12, with higher scores indicating heavier alcohol assumption. For men, they met criteria for the substance misuse stressor if they received a score of 4, whereas for women, they met the criteria if they received a score of 3 or more.

**Drug use.** The Drug Abuse Screen Test [34] is a 10-item measure that assesses drug-use related problems within the past year. Participants answered “yes” or no” for each item. A score of “1” is given for a “yes” response except for Item 3 (“Are you always able to stop using drugs when you want to?”). Items are summed for a total score, with a score of ≥3 meeting criteria for the drug use stressor.

**Demographic variables.** Parents provided demographic information, including monthly income, household size, gender, ethnicity, race, education, employment, and marital status. Financial strain was defined as a household income ≤ 200% of the federal poverty guideline.

#### 2.3.2. Survey Measures

Eligible parents completed the following measures assessing child well-being, parent well-being, and parenting practices. All measures demonstrated adequate reliability (see Table 2). See Table S1 for sample items.

**Child social–emotional functioning.** The Strengths and Difficulties Questionnaire (SDQ) [35] consists of 25 items that measure emotional and behavioral problems. The SDQ is composed of five scales: emotional problems, conduct problems, hyperactivity problems, peer problems, and prosocial behavior. Participants rated each item on a scale of “*Not true*” to “*Certainly true*.” Items from all but the prosocial scale are summed for a total difficulties score. Items from the conduct and hyperactivity problems scales are summed for an externalizing score, and items from the emotional and peer problem scales are summed for an internalizing score. Higher scores indicate more difficulties on all except the prosocial scale, in which higher scores indicate better prosocial functioning.

**Child lifestyle behaviors**. For physical activity, participants responded to a question assessing their child’s physical activity level excluding school time on a 5-point Likert scale ranging from “*Very low*” to “*Very high*” [36]. Sleep was measured using the 5-item BEARS screening tool [37]. Participants responded “yes” or “no” to items related to bedtime issues, daytime sleepiness, and regularity and duration of sleep. Screentime was assessed by averaging two items about quantity of screentime on weekdays and weekends.

**Parenting practices.** The 34-item Multidimensional Assessment of Parenting Scale (MAPS) [38] was used to assess positive and negative dimensions of parenting practices over the past two months. Participants rated each item on a 5-point scale from 1 = *Never* to 5 = *Always*.

**Parenting stress.** The Parenting Daily Hassles Scale [39], Challenging Behaviors subscale, contains 7 items that could be a stressor to the parent. Items are rated on a 5-point scale from 1 = *No hassle* to 5 = *Big hassle* and are totaled with a higher score indicating more of a hassle.

**Parental self-regulation**. The Parenting Self-Regulation Scale [40] assesses perceived competence and efficacy as a parent and consists of 12 items on a 7-point scale from 1 = *Strongly disagree* to 7 = *Strongly agree*. The total score was calculated by summing all items, with higher scores indicating higher levels of parental SR.

**Parent psychological well-being.** The depression, anxiety, and stress scale (DASS-21) [41] is a 21-item measure that assesses the emotional state of depression, anxiety, and stress. It is a short version of the full 42-item DASS and has three subscales with 7 items in each subscale (depression, anxiety, and stress). Participants rated each item using a 4-point scale ranging from 0 = *Did not apply to me at all* to 3 = *Applied to me very much or most of the time*. A higher score indicates higher severity.

### 2.4. Qualitative Interviews

Parents participated in individual interviews that explored their perceived strengths and challenges and the impact of stress on their own and their child’s well-being. Interviews were conducted by the first, second, and sixth authors, all of whom had clinical training and experience with qualitative interviewing. Example questions included, “What are your main sources of stress?” and “What are the strengths that you draw on to deal with life stress and challenges?” Interviews also included questions about intervention opinions and preferences, such as “If you were designing a parenting support program, what kinds of topics or strategies do you think would be most helpful for parents who face life stress?”.

### 2.5. Data Analyses

Quantitative analyses were conducted using R and SPSS 28. Descriptive analyses included examination of participant demographics, means, standard deviations, and bivariate correlations. These analyses were conducted to characterize the overall child and family functioning of the sample and to explore how key study variables were associated. Given the sample size limitations and descriptive nature of this study, multivariate analyses were not conducted. Qualitative interviews were transcribed verbatim and analyzed using a directed content analysis approach informed by grounded theory [42]. This approach involves a combination of deductive and inductive coding. Some a priori domains were proposed based on theory. For example, a priori domains included family strengths (based on resilience theory) and effects of stress on families (based on the family stress model and process model of parenting). Within each of these domains, inductive themes emerged during the coding process. The codebook was created in a collaborative, iterative process. NVivo (version 12) was used to organize and conduct the qualitative analyses. The qualitative coding team included three coders: first, second, and fourth authors. All coders had expertise in qualitative methods, child development, and parenting and family processes. Each interview was coded by two coders, and meetings were held to reach consensus on the codes for each interview. Any discrepancies were discussed until consensus was met. We evaluated data saturation by assessing the number of new codes being added with each interview and discussing whether each theme and key concept was fully described in the interviews. We assessed that data saturation was reached for all themes, given quality, consistency, and richness of the results, and used this evaluation to guide decisions to cease data collection and report findings.

## 3. Results

### 3.1. Quantitative Results

#### 3.1.1. Parent and Child Well-Being Indicators

Descriptive results are displayed in Table 2. In line with our objective of describing children’s social–emotional and lifestyle health behaviors in families facing stressors, the findings demonstrated that parents, on average, reported slightly elevated total difficulties in their children (45.65% above clinical threshold), moderate levels of child externalizing behaviors (47.83% above clinical threshold for conduct problems; 32.61% above clinical threshold for hyperactivity), low to moderate levels of child internalizing symptoms (30.43% above clinical threshold for emotional problems; 17.39% above clinical threshold for peer problems), and moderate levels of child prosocial behavior (43.48% above clinical threshold). Parents reported an average of 1.72 (out of 5) child sleep problems, an average of 112.04 (SD = 71.60) minutes for child physical activity during the weekdays, an average of 121.60 (SD = 92.73) minutes for child physical activity during the weekends, and an average of 2–3 h of child screen time throughout the week and on the weekends.

As part of our broader goal to further understand parenting practices and parental well-being, especially for those facing adversity, findings suggested that parents, on average, reported high levels of positive parenting and low levels of negative parenting. Parents reported moderate levels of parental SR and moderate levels of daily hassles regarding the child’s challenging behaviors. Lastly, parents on average were in the mild to moderate clinical range for depressive symptoms, anxiety symptoms, and stress.

Bivariate correlations are displayed in Table 2. Overall, child social–emotional difficulties were significantly positively related to negative parenting, parenting daily hassles, parent depressive symptoms, anxiety symptoms, stress, and child sleep problems and negatively related to positive parenting. Child internalizing symptoms were significantly positively related to parent depression, anxiety, stress, and child sleep problems and were negatively correlated with parental SR. Child externalizing behavior was significantly correlated with more negative parenting, parent daily hassles, parent depressive symptoms, and parent stress. Child prosocial behavior was associated with more positive parenting and less parent stress. Negative parenting was related to more parenting daily hassles, parent depression, anxiety, stress, and child screen time. Positive parenting was related to higher parental SR and lower depression and stress symptoms. Parental SR was negatively related to both depression and stress. Parent depression, anxiety, and stress were each related to more child sleep problems. Parent depression was also related to less weekend physical activity for children. Otherwise, no other study variables were associated with the parent report of child physical activity.

We also conducted independent samples t-tests and chi-square tests to explore possible differences between the qualitative interview subgroup and the quantitative-only group. No significant differences were found in parent or child sociodemographics. However, the quantitative-only group was significantly more likely to use physical control as a parenting practice compared to the qualitative subgroup, *t* (43.00) = −0.50, *p* = 0.620; *d* = −0.148.

#### 3.1.2. Parenting Support Program Preferences

**Figure 2:**
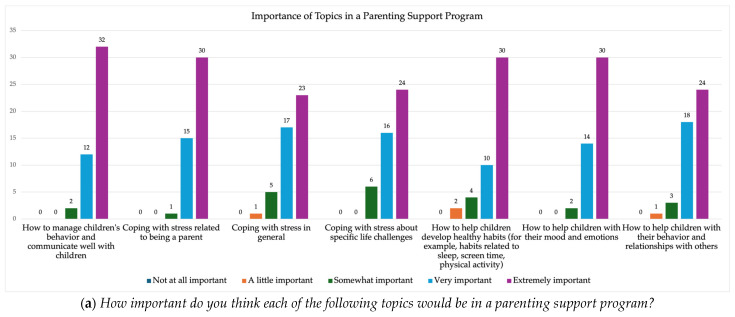

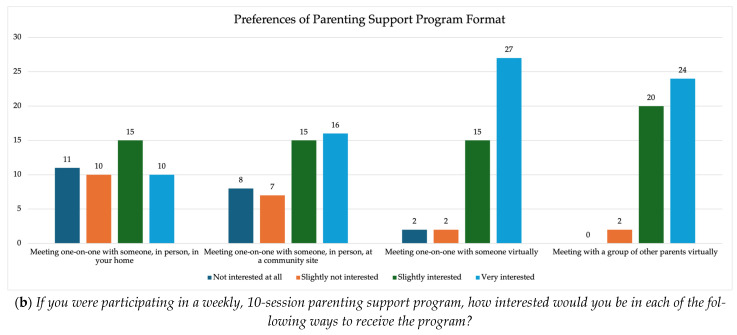
Results of survey items regarding parenting support program preferences.

The vast majority (93.5%) of participants were very or slightly interested in participating in a parenting support program. Most (56.5%) believed that other parents would be interested, while 41% were unsure. Parents believed that the most important topics included how to manage their child’s behavior and communicate well with children, coping with stress related to being a parent, how to help children develop healthy habits (i.e., related to screen time, physical activity, and sleep), and how to help children with their mood and emotions (Figure 2a). When asked about modality preferences, parents expressed the greatest interest in one-on-one and group virtual delivery (Figure 2b). Moderate interest emerged in in-person individual delivery at a community site. Parents were least interested in one-on-one in-person delivery at home.

### 3.2. Qualitative Results

Seven themes, each with its own sub-themes, emerged from the parent interviews: family strengths, child strengths and challenges; parent stressors, effects of stress on families, parent strengths and strategies to manage stress, helping children deal with stress, and parenting support program preferences and opinions. Table 3 summarizes all major themes and subthemes with one representative quote for each.

#### 3.2.1. Theme 1: Family Strengths

Nearly all parents (96%) described family strengths. The most frequently discussed family strengths were family values, closeness, and unity (52%) and communication (30%). Parents also identified strengths of having independence, perseverance, and flexibility (22%); having extended family support (17%); and being loving and affectionate (9%). A parent of a 6-year-old emphasized quality time together by saying, “*One of our strengths is that we enjoy doing things together. We go on adventures and just enjoy the stuff we do”* [ID 7]. Several parents discussed communication about emotions, allowing children to express themselves, and having a sense of closeness. A parent of a 4-year-old identified communication and forgiveness as a family strength:

*If I make a mistake, I make it known, “Mommy shouldn’t have gotten upset or yelled or said that. You did do wrong, but Mommy was wrong, too.” I don’t pretend to be perfect. So that comes out as a strength, because I’ve also had it to where now she comes to me, ‘cause that’s been going on a year and a half, two years, ‘cause I started it early. She’ll come to me now after she’s calmed down and stuff and apologize. I don’t know. I think it makes her more comfortable to approach me.*

[ID 12]

Some parents discussed characteristics of their family that are both challenges and strengths. For example, a parent of a 7-year-old said the following:

*I’m a single mom. My daughter is seven. I do have a lot of support from both of her grandmothers, but I would say that I guess independence is a strength, if you want to call it that. We do the best we can and we get by. I don’t receive any kind of child support or anything like that. So in my eyes it’s a strength, but at the same time it definitely creates, I guess, some struggles from time to time. But I guess the perseverance is a strength.*

[ID 24]

A few parents discussed strengths that contrasted with their own upbringing. For example, one parent of a 7-year-old said, “*I never when we grew up in my household had people tell you ‘I love you,’ so I do it to my kids all the time. And so, they’ll just be like ‘Mama, we love you.’*” [ID 34]. Another parent of a 5-year-old similarly described an intergenerational shift by stating the following:

*I think that we are really affectionate, and I think that we are really great about communication. We’re really good about trying to talk about our feelings or just be a bit more communicative than probably most of our parents were.*

[ID 9]

#### 3.2.2. Theme 2: Child Strengths and Challenges

In describing children’s characteristics, all parents (100%) identified strengths in their children, primarily falling in the domains of personality traits (70%), academic skills and intelligence (48%), hobbies (17%), and interpersonal skills (13%). Parents described their children using terms such as *friendly, outgoing, outspoken, caring, sweet, funny, active, smart, and passionate:*

*He is very curious about the world around him. He always asks questions. We’re always laughing at him … I think his curiosity really drives him to discover how things work. He likes to know how things work, how the world works. So I think that’s probably going to take him pretty far.*

[parent of a 5-year-old; ID 21]

*Oh, she’s very smart. She has really good comprehension skills … it also helps me with discipline, because…I see that when I talk to her, it’s easy to have conversations with her and talk things through to her, because I really know that she understands me, and then she gives me feedback to show me that she does understand me.*

[parent of a 3-year-old; ID 16]

Challenges identified by parents regarding their children included challenges with peer relationships, emotion dysregulation, the child’s trauma history, and academic or school challenges:

*He does have a temper and gets very frustrated with things and will yell out or lash out when he’s angry, so that’s something that we’ve been trying to work on and help him with.*

[parent of an 8-year-old; ID 4]

*If he’s tired or there’s a lot of people around and there’s a lot going on, then he can start acting really hyper and crazy. So, a lot of our challenges have just been around like, how to diffuse some pressure and chaos and heightened excitement when he gets there.*

[parent of a 6-year-old; ID 3]

#### 3.2.3. Theme 3: Parent Stressors

All parents described one or more stressors. When asked about primary stressors in their life, parents most often discussed parenting-related challenges (83%), financial strain (96%), competing demands (e.g., work and parenting; 65%), and challenges with other family members (70%; e.g., coparenting, caring for aging relatives, lack of family support). Additional stressors included parents’ mental health, trauma history, physical health or HIV, and experiences with racism and discrimination. Nearly all parents discussed financial strain as a major stressor:

*My main source of stress with my children would have to be money, I guess. And keeping up with the bills.*

[parent of a 4-year-old; ID 43]

Almost all parents described challenges related to parenting, such as being a single parent; concerns about children’s nutrition; childcare concerns; and dealing with children’s developmental, mental health, or medical difficulties. Most parents described the stress of balancing competing demands, including parenting responsibilities, work, and other obligations. For example, a parent of multiple children noted that a major challenge is “*finding the time to spread between all three children. It’s pretty time consuming, I would say, trying to do that. And then balance social life and work life and everything”* [ID 27]. Many parents discussed their own mental health, psychological well-being, and past trauma as sources of stress:

*After I had my daughter, I had never experienced anxiety or stress a lot, but I actually ended up developing an anxiety disorder. I don’t think that it was necessarily from my children, but I do think that I have a much higher tendency to get anxious feelings now than I ever did before—for so many reasons, including postpartum, being worried about your kids—just so many different things. And I think that’s probably one of the major differences or things that I’ve seen that has come from the extra stress that you don’t have when you’re not a parent.*

[parent of a 5-year-old; ID 9]

*A lot of my past really does come into play, especially the relationship with my parents comes into play almost daily. It’s really horrible how often it comes into my life because it’s so distracting and negative. So, I mean, I feel like it’s all self-inflicted, maybe years of suppressing certain feelings or certain thoughts to try to get through hard situations. Like, a situation I feel like time just had to pass to get through it. So, kind of blocking negative things. So, that’s a source of stress.*

[parent of a 5-year-old; ID 41]

A few parents also described their experiences with substance use and parenting:

*I used to drink a lot more and I noticed some really unhealthy behavior patterns and I noticed that I would drink a little bit too much. Not like a ridiculous amount but have a couple drinks when [Child] was awake or around and I didn’t like the way that I felt. I didn’t like the way that I felt the next morning. I didn’t like having a headache and trying to interact with my child.*

[parent of a 5-year-old; ID 21]

*I’ve gotten help for [my drug use] now and things are a lot better now. But when you’re addicted to a substance or something like pain pills, you’re always worried about …am I going to be able to go to work when I’m in this much pain?*

[parent of a 4-year-old; ID 12]

Experiences with racial discrimination were discussed by a few parents (35%). For example, a Mexican American parent of a 5-year-old said, “*As a parent, in the community, [discrimination] can make you feel less important. Sometimes, that can be a knock on your confidence … it can kind of make you put labels on yourself that maybe others have*” [ID 9].

In some instances, parents described how multiple stressors compounded one another. For example, a parent with multiple children described the cascading and reciprocal effects of childcare costs, employment, and COVID-19 impacts:

*I’ve turned down better paying jobs because of not having childcare. And the cost … because of their ages, they’re both in the most expensive rooms at daycare … And, at one point in time, even with COVID and everything, they shut down some classrooms. So, at one point in time, his daycare didn’t have a one-year-old teacher so, he had no choice but to stay in the infant room, which is the most expensive room.*

[ID 16]

#### 3.2.4. Theme 4: Effects of Stress on Families

All participants were asked to reflect on how their various stressors impact them as parents, and subthemes emerged related to the impact of stress on the child’s well-being (social–emotional, physical, and academic), the impact of stress on parenting and parent–child interactions, and the impact of stress on parents’ own emotional well-being and physical health, as well as ways that stress does not have a major impact.

**Impact of stress on child well-being**. Eighty-three percent of parents discussed the emotional, behavioral, and physical well-being effects of stress on their children. Emotional and behavioral effects were most discussed (74%), with many parents explaining that their stress is often reflected in their child:

*There’s been … a couple times where I’ve just kind of lost it a little bit and he’s gotten upset. And then I end up crying, too, because I feel awful … I worry that if I don’t keep my stress under control, that he won’t want to come to me with problems he’s having because he doesn’t want to get that reaction. So I really try to keep that under control.*

[ID 21]

*I also noticed that when I’m upset especially, or having just stressful times, she’ll not be nice to her younger brother, which is hard to witness, because I can see a lot of the things that I do and say, or the ways that I treat her, she’ll treat him. That’s a pretty good mirror, I’d say.*

[ID 41]

Several parents described changes in their child’s physical activity level and/or screen time as an effect of their own stress (35%). For example, the parent of a 5-year-old said, “*When I’m sad or like just having an overwhelming day I notice that he’s less moving around and jumping; he just kind of doesn’t act the same*” (ID 26). Parents described how they adjust the child’s screen time based on stress level:

*I might increase [screen time] or I might decrease it—increase it if I’m dealing with something to give me a minute to decompress. But I might cut it off early so I can go ahead and put him down, so he can go ahead and go to sleep so he don’t see the stress.*

[parent of a 7-year-old; ID 10]

*I think that when I’m stressed, I’m probably more preoccupied with working on whatever that stress factor is or trying to become unstressed that we tend as parents to be like, “Hey, why don’t you watch TV or play on your tablet for a few?” so we can gain those little moments to try to recoup ourselves.*

[parent of a 4-year-old; ID 22]

**Impact of stress on parenting and parent–child interactions.** Nearly all parents (96%) discussed ways that their stress negatively impacts their interactions with their child, such as reduced patience, withdrawing, or spending less time with the child:

*I feel like you can get frustrated more easily or be more distracted, and I feel like it kinda takes away from your patience, in some ways, your presence of how present you really are with your child in that moment… And regardless of what anybody says, they pick up on that, too. They can tell when you’re fully engaged versus when you’re partially.*

[parent of a 4-year-old; ID 12]

*I have this ideal vision of how I would want to be as a mom, and it’s someone who is really engaged and doing fun things with my kids and like—yeah. Snuggling and talking about their day, and I frequently don’t feel up to that.*

[parent of a 6-year-old; ID 2]

Several parents (65%) expressed that financial strain makes it difficult for them to provide their children with material goods or activities:

*There’s so much I want to do for them, so much I want to provide for them and I can’t because financially I’m not able to.*

[parent of a 7-year-old; ID 10]

*I’m more agitated. So, with my parenting everything they do now seems to bother me ten times more because I’m always stressed out—how many more hours I need to work or what double can I pick up to make sure these bills are paid, and then I need to be able to make extra money to be able to do something with them or keep them from being cooped up in the house. So, it’s really hard sometimes.*

[parent of a 5-year-old; ID 47]

A few parents described ways that racial discrimination as a particular stressor affects them:

*It [racial discrimination] might affect the way that I want to go about protecting her and how I engage with other people who I know are gonna be engaging with her, then it makes me scrutinize situations a little more or be more particular about who I have her around or how I handle them so that they know that I am an involved parent and don’t treat her any old kind of way, ‘cause I will say something. So, I think it…makes me more defensive, and kind of more anxious, because I don’t want them to have bad experiences. And I know that sounds out of my control, but if it’s something I can do to control it, then I try.*

[parent of a 3-year-old; ID 16]

*I was the only African American at the office and at times, I did feel, you know, some differences and things like that. Sometimes you come home, and you’re drained from having to deal with all those things and the interactions and all of that. And also, to keep a positive attitude to pass on to your children. So that was a frustrating time.*

[parent of a 9-year-old; ID 30]

Parents also described ways they use stressful moments as opportunities to teach children about emotions:

*She can read stress on me because she’ll say it and she communicates it…She’ll ask me if I’m okay. She’ll ask me if I’m mad. She’ll ask me if I’m happy. She’ll ask me if I’m frustrated. Like, she says those words. And…the more I’ve noticed different emotions that I’ve expressed in front of her, I try to use different words to help her understand emotions. So, I try to use words like, “No, [Child], you were disappointed. We didn’t do whatever that was that you wanted to do, so what you’re feeling is disappointed. But we’re gonna be able to do it another time. But, right now, you’re disappointed, ’cause we couldn’t do it right now.” And I notice when I use certain words like that with her, if she feels like I’m experiencing it, she’ll say it to me like, “Are you disappointed? Are you frustrated?” So I think that’s more of a positive way that the stress affects her, because she’s able to explain it.*

[parent of a 3-year-old; ID 16]

**Impact of stress on parent well-being.** Parents described both emotional (91%) and physical (70%) effects of stress on their well-being. One parent of a 7-year-old mentioned “*lack of sleep, lack of focus, irritable, moody*” and others mentioned “*my patience,” “frustrated more easily,” “more defensive,” “more anxious,”* and *“headaches,”* as ways stress affects them:

*Oh, like I’m tense all the time. Like my jaw hurts right now. It’s hard to find time to take care of myself. I’ve lost a lot of friendships because I just can’t hang out the way that other people do. It’s put a lot of strain on my marriage. Clearly I need to see a therapist because like just all these emotions coming up the first time. Like I don’t talk about this stuff often. It’s like I really keep bottled up because it feels so socially unacceptable to talk about how hard it is.*

[parent of a 6-year-old; ID 2]

#### 3.2.5. Theme 5: Parent Strengths and Strategies to Manage Stress

Parents shared a variety of strengths and strategies to help them manage life stress and challenges, including social support (39%), religion/spirituality (13%), and hobbies or enjoyable activities (39%). Other strategies included emotion regulation (22%) and problem solving (17%). Several parents expressed that having social support from others, such as their partner, family, or other parents, has helped them cope with daily life challenges and stress. A parent of a 4-year-old shared the following:

*I have a husband who helps me. I have a sister. I have a village. So it’s not like me by myself. But sometimes I feel like I’m by myself. But they help me see things in a positive way.*

[ID 17]

A few parents described how their faith/spirituality has been a strength to cope with stress. For example, a parent of a 9-year-old said, “*I was raised in the church, so I have a spiritual foundation that keeps me grounded*” [ID 30], and another parent said, “*You don’t have to stress about the little things, as far as a bill or something because I have—I’m a religious person, so I believe in God. So, I believe I do my part; he does his. It always tends to work out*” [ID 34]. Some parents described engaging in hobbies or enjoyable activities to help manage stress. For example, a parent of a 7-year-old said they like to “color” and “cook.” Another parent of a 6-year-old said the following:

*We love going hiking out in nature. I am all for re-grounding. Nature is your friend. Nature will give you what you need… We go to as many [state parks] as we can or we just go for a walk. Just go for a walk. That’s our thing.*

[ID 7]

Parents also shared using emotion regulation and cognitive coping strategies, such as deep breathing or reframing the situation:

*I’ve been doing something that really helps. So when I’m confronted with a stressful situation or something that doesn’t go my way, instead of being reactive to it, I say that’s so silly. Like oh, that’s so silly, our plans changed. And I feel like just in saying that it makes me—you can’t then be super tense. When you say silly, it makes your face smile and you feel relaxed. So that’s kind of a new thing that I been trying that really helps.*

[parent of a 5-year-old; ID 21]

*There’s just so much that I’m so worried about. But I’m just trying to learn new ways to cope and breathe and just say “Everything’s going to be okay,” because I do try to do my best. I just try to take it one day at a time.*

[parent of a 7-year-old; ID 34]

Parents were asked about how effective their strategies were. Most parents expressed that their strategies “worked well”, while others described that they work some or most of the time:

*It works, you know, I’d say maybe 80% of the time. Then there’s days where it’s just like, as they say, all hell breaks loose and it’s just one thing after another. And those are things we can’t control. So, it’s just you know, trying to be positive and strong to you know, finish the day out and look forward to the next day.*

[parent of a 9-year-old; ID 30]

Another parent of a 5-year-old expressed that it was challenging to engage in strategies to help cope with stress due to competing stressors:

*I would say probably about 75%, because I can’t do it every day. I can’t model every day because I have a full-time job. I can’t take my kids out every single day because I don’t have the funds, and then I can’t talk to my therapist every day because she has other people, so I would say about 75%.*

[ID 35]

#### 3.2.6. Theme 6: Helping Children Deal with Stress

Parents described numerous ways they help their children manage stress, ranging from communicating about stress and emotions (61%), facilitating children’s use of emotion regulation strategies (57%), using active play and enjoyable activities (30%), guiding the child through problem solving (26%), modeling resilience (13%), providing positive reinforcement (13%), giving physical affection or comfort (17%), and drawing on community resources such as therapy (4%). One parent of a 6-year-old mentioned using a variety of strategies:

*We talk about what’s stressing him out. We ask a lot of questions about what goes on at school, how he’s feeling. Sometimes, it’s physical touch; it’s a big bear hug. We help him practice deep breathing. Sometimes, it’s going and running laps.*

[ID 3]

Several parents described communication as a key strategy, such as the parent of a 5-year-old who described instilling the value of emotional expression: “I’m trying to promote that having a calm mind and being able to express your feelings is a healthy thing” [ID 47]. A parent of a 9-year-old mentioned drawing on religious/spiritual practices: “I try to pray with my children and give them words of affirmation every morning” [ID 30].

Other parents described physical activity, being outdoors, and active play as stress-management strategies for their children, such as the parent of an 8-year-old: “*He’s very active, so I think when he sits and plays a videogame instead of playing outside, I think he gets more stressed out, so we kind of limit screen time and encourage more outdoor activities*” [ID 4].

In reflecting on the effectiveness of the strategies they employ, most parents felt they work well, such as one parent of a 6-year-old who said, *“I can see a difference from a couple years ago when we weren’t quite using as many of our strategies as opposed to now where we have different strategies that are there”* [ID 7]. Another parent of a 5-year-old said, *“I think they’re really helpful. We’re never going to reduce stress all the way and for a child, they are growing and changing so often. But I think it really helps”* [ID 21].

Other parents, however, described struggling to know how to effectively help their children deal with stress. One parent of a 4-year-old reported, “*It’s not working at all*.” Another parent of a 5-year-old said the following:

*I don’t really know how to help them, actually help them. So, I would probably do the wrong thing. Like, “Don’t worry about that.” Yeah, they’re clearly worried about that. “Don’t worry about that.” That’s something that’s not helpful. “And now, take a deep breath.” I don’t know. That’s doesn’t seem to help me. Or maybe I just don’t think it will help. It doesn’t seem like the right answer, but that will be an answer sometimes—it will be a response that I’ll say sometimes.*

[ID 41]

Similarly, another parent stated the following:

*Sometimes it’s harder to recognize what exactly is triggering him, because sometimes they don’t know at that age. So it’s kind of hard to know, too. And with my daughter getting extra-large emotions lately and working through them, which I guess is typical, sometimes I notice the struggle of trying to understand what’s wrong, what it is she needs in that moment.*

[ID 12]

#### 3.2.7. Theme 7: Parenting Support Program Opinions and Preferences

When asked to generate topics that would be most valuable to include in a parenting support program for parents experiencing life stress, the most frequently mentioned topics or recommendations related to community resources (35%; e.g., connecting with existing financial assistance resources), building up social or peer support (30%), helping children with emotion regulation (30%), and psychoeducation about child development (26%). Parents suggested incorporating content on “*ways you reach out into your community and find out what your community can offer*,” “*what children should be doing at certain ages,*” “*time management*,” and “*how to explain more in a child’s way.”* One parent of a 6-year-old suggested incorporating realistic strategies for implementing positive parenting practices:

*There are going to be a lot of parents who already have the information. They need help figuring out how to connect strategies and connect resources and think about, “Okay, this is all really hard, but with what I have what could I change? What can I tweak?” And to me that would be the thing that would be the most helpful.*

[ID 2]

Parents were also presented with potential topics and asked to share how useful or helpful they believed each one would be. Regarding positive parenting, most parents indicated they would find this topic very useful:

*I’ve got a lot of life stress right now, and sometimes I get more stressed when I can’t figure out what she needs, ‘cause it’s not always that simple, and it’s hard to help her respond to something she’s thinking or feeling if I don’t know what it is or I don’t know the best way to deal with it.*

[parent of a 4-year-old; ID 12]

*I think there’s a lot of people who know that there’s better ways to handle it, but they just don’t know the better ways or just don’t know how they can implement it within themselves.*

[parent of a 3-year-old; ID 16]

Most parents expressed interest in a program with a dual focus on children’s social–emotional and lifestyle behavior health, including content related to healthy eating, bedtime, and active play. Parents described the potential value of including topics such as “*nutrition and healthy snacks,” “setting a bedtime,” “sleep,” “taking your child outside,”* and *“understanding the importance of those healthy habits and why eating healthy is so important*.” Regarding screen time and sedentary behavior, one parent highlighted that “*we’re definitely in a time where the easiest thing to do is hand them a phone as opposed to sending them outside to play*.” Another parent emphasized the need to address barriers to supporting healthy lifestyle behaviors:

*We know screen time limits are good, we know kids should be playing outside, so it’s important to dig into why that’s not happening. Like for example, [Child] watches more TV than I feel good about, but the reason that she’s watching TV is because I can put her in her room with the TV while I take a shower, and it keeps her safe. Or when I’m making dinner. And if I had more support in my life, it wouldn’t be maybe necessary in the same way… I think for the vast majority of parents it’s not a lack of knowledge; there’s a reason that we’re doing and not doing these things and there are barriers there.*

[ID 2]

With respect to topics that deal with parents’ own well-being and mental health, such as coping with emotions, practicing self-compassion and mindfulness, taking care of adult relationships, dealing with the past, and forming healthy habits, most parents expressed that they believe these topics would be relevant and helpful to them.

*I would say as far as the topics, let me focus on mental health as a parent, and also focus on the health of the child.*

[parent of a 7-year-old; ID 27]

*I have a lot of past trauma in my life that I have yet to deal with, and a lot of them are negative, and I don’t know how to let it go. And I’m afraid that my kids knowing about some of the negative things I went through will have an effect on their life, because I watched certain things with my mom and I feel like I’m going through similar things that she went through, so the cycle is just—it’s being passed on and I want to know how to break it.*

[parent of a 5-year-old; ID 47]

*I think supporting and strengthening [the coparenting] relationship is probably one of the top things that can help parents who are really struggling.*

[parent of a 6-year-old; ID 2]

A few parents described a potential pitfall of addressing parental self-care at a superficial level:

*My caution on that topic…is self-care can’t be this Band-Aid so that you can continue doing more than you’re actually capable of. It has to be a deeper thing about boundaries and things that actually long-term that are healthy.*

[parent of a 6-year-old; ID 2]

*I feel like we all hear about self-care and it’s like oh, go take a bubble bath or get your nails done. But what it really should be is learning how to be mindful and set boundaries and do deep breaths.*

[parent of a 5-year-old; ID 21]

Some parents expressed mixed feelings about topics related to parents’ past life events:

*I think it depends on the scope of the group and how much time—like the other things I feel like would be more, not more relevant but more like a higher priority. But I feel like if it was an ongoing group and there was time to talk about that kind of thing, I think that would be important. But not as important as learning to communicate with your child and emotions and that kind of thing.*

[parent of a 5-year-old; ID 21]

In contrast to most parents, a few parents shared the view that topics related to taking care of adult relationships would be less relevant to them:

*That [topic] doesn’t sound interesting to me, I don’t think. Yeah. I wouldn’t be interested in that.*

[parent of a 6-year-old; ID 3]

*It might be more useful for other parents. It’s not really an important topic for me…At this point like I don’t know, dealing with the relationship, friendships, and things like that is not an important thing for me. I just choose to have other things that I’m just more concerned about right now than that.*

[parent of a 4-year-old; ID 43]

When asked about their preferred modality (e.g., in-person vs. virtual delivery), parents raised a number of potential benefits and drawbacks of each modality. Many parents expressed that flexibility should be offered so that individual parents can choose the modality that works best for them, such as one parent of a 7-year-old, who said, “*There should be options for everybody, for those that don’t have transportation or childcare”* [ID 10]. Parents who preferred virtual delivery cited reasons related to convenience, privacy/trust, or COVID-19 concerns. Several parents expressed that the convenience of virtual delivery may help overcome a primary barrier—time constraints:

*I think the best option for most people would be probably remote—like, on Zoom or something like that…because of the time restraints. I feel like most people have to work at least 40 hours a week, and if you are getting home later, you don’t have a whole lot of time in the evenings to do what you need to do and extracurricular activities, that’s even more stuff on your plate. So, I think, really, it’s just a lack of time.*

[parent of a 5-year-old; ID 9]

*I think the challenge, for at least the people I know, is time, is like when we would do it. Like if it was in the same time, like a live something, that’s harder than something that we could do on our own and at a self-paced way.*

[parent of a 7-year-old; ID 56]

On the other hand, some parents expressed that in-person formats facilitate more engagement:

*I think remote is the most convenient for everyone, but it’s also really easy to blow off when it’s remote. It feels a lot less like a commitment.*

[parent of a 6-year-old; ID 2]

*I feel like probably online would be best for me because then I don’t have to go anywhere and do anything. But also, you lose the face time, the communication. It’s great online, but the personal interaction is just not possible. Especially if it was a group, it’s very hard to chat with other people or even just interact. So, I don’t know. Meetups would be good. But I feel like I would be interested in doing work remotely also. Okay, maybe it would even make some of the more difficult things easier, like “Talk about your past.” Oh, God. Doing that in person could be very uncomfortable compared to online.*

[parent of a 5-year-old; ID 41]

## 4. Discussion

Guided by the process model of parenting [14], the family stress model [3], and resilience theories [21], this study used mixed methods to explore parents’ experiences with major stressors, the perceived impact of those stressors on family well-being, and preventive intervention preferences. Quantitative and qualitative results revealed a moderately high level of stress with some variability regarding the number, severity, and type of stressors faced by families. Consistent with prior research showing high co-occurrence of the kinds of stressors examined in this study, over two-thirds of parents experienced three or more major stressors, and several participants had five of the six stressors examined. Among eligible participants, the most common stressors were financial strain, traumatic event history, and mental health concerns. This was reflected in the qualitative interviews in which financial stress was a common theme across domains. Many parents also discussed their psychological well-being and trauma history as major stressors affecting their parenting and family well-being. Underscoring the relevance of a syndemic conceptualization [17], several parents described compounding effects of multiple stressors (e.g., childcare, employment, pandemic-related concerns). Quantitatively, parents reported moderate to high stress levels, with many parents in the clinical range for psychological distress. Across different stressor types, there were qualitative commonalities regarding how stress relates to children’s functioning, parent–child interactions, and parents’ own well-being. Consistent with the family stress model and process model of parenting, most parents discussed ways their own stress leads to emotional and/or behavioral dysregulation in their children, less frequent or engaged parent–child interactions, fewer activities together, and both physical and emotional symptoms in parents. These themes converged with the quantitative findings, revealing associations between parental well-being (depression, anxiety, stress, parenting-related stress) with child outcomes and parenting practices. Though this was a community-based sample, children’s emotional and behavioral difficulties were somewhat elevated, consistent with prior research that children in families experiencing adversity are more at risk for adjustment difficulties. Overall, these findings are aligned with the prior literature on the effects of stress on families [3] and extend these results to a sample of families experiencing multiple, co-occurring stressors.

A contribution of this study is the emphasis on protective factors and families’ abilities to cope with stress, drawing from resilience theories [21]. Parents described a range of strategies that they employ for themselves or their child to manage stress, including problem-focused coping techniques such as positive communication, problem solving, and seeking social support, as well as emotion-focused coping techniques such as emotion regulation, cognitive reframing strategies, and physical activity. Fortunately, many parents expressed a sense of efficacy in employing and benefitting from these strategies. This indicates an area of strength in that parents affected by major stressors draw on a range of factors to promote their own and their children’s resilience. At the same time, several parents expressed difficulty with finding effective coping tools, and even those who had effective strategies still described extensive negative impacts of stress in their lives. In the context of healthcare services for families, additional supports may complement and further bolster parents’ ongoing stress management efforts to promote positive developmental outcomes for children. These findings align with recent efforts to expand family theories, such as the family stress model, to incorporate a stronger focus on resilience and protective factors [43]. Understanding both the effects of stress on families as well as the protective factors on which families rely is critical to providing culturally relevant, strengths-based services.

Parents expressed a strong interest in receiving a parenting support program with an integrated focus on positive parenting strategies and parental stress reduction. Both qualitatively and quantitatively, parents expressed interest in gaining practical skills for positive parenting and skills to address their own stress and well-being. Parents’ willingness to address their own sources of stress in the context of a parenting program indicates a promising path of integrating parental emotional skills and self-regulation into programs and healthcare services that are traditionally child-focused. An integrated preventive intervention may be a more efficient means for parents with heightened stress to address concerns related to their child and their own well-being without seeking separate services, particularly given that adult mental health services typically do not address parenting-related concerns [44]. These findings connect with intervention studies that target parental stress in other high-risk groups, such as parents of children with medical illness [45].

Parents expressed interest in intervention content focusing on dual domains of child social–emotional and lifestyle behavior health. Several parents generated lifestyle behavior-related topics, such as child nutrition and sleep, as priority areas. Parents discussed linkages between their own well-being and their child’s lifestyle behavior, such as allowing more screen time or facilitating less active play when stressed. These findings underscore that parenting and parental well-being are influential for both children’s social–emotional health and physical health. Thus, addressing parents’ stress in a preventive intervention and enhancing parenting skills that target both domains is a promising avenue for promoting children’s overall well-being and promoting equity for families experiencing economic and racial marginalization. Preventive intervention in this population could also be enhanced by acknowledging differences in cultural norms across households, considering varying experiences with racial discrimination across the life course, and assessing the impact of intergenerational trauma on parental functioning and parenting, particularly in multigenerational households where grandparents are contributing to child-rearing [16,46].

Regarding modality, there was a strong preference toward virtual delivery, including both individual and group formats. This preference may also have been influenced by the fact that data were collected through online surveys and virtual interviews and perhaps also reflected shifts following the recent COVID-19 pandemic, coupled with broad shifts to more technology-based services [29]. Parents’ expressed desire for flexibility and tailoring was consistent with prior research on parent intervention preferences [28].

### 4.1. Limitations and Strengths

An important limitation of this study is the participants’ heterogeneity in stressor type and severity. Though there are benefits to sampling a relatively broad group of parents experiencing stressors, a tradeoff is the limited ability to draw conclusions about specific stressor types and the mechanisms by which they relate to outcomes. Further, given the small sample size and reliance on parent report, findings in this study (e.g., rates of stressors observed) may not be generalizable to the broader population and need more robust sampling and measurement in future studies with larger samples and multiple informants. Additionally, although this study was open to caregivers of any gender, only female participants enrolled; the important role of fathers warrants further examination. Another limitation is the coarseness of the stressor screening measurement. Trauma exposure, for example, was measured using a single yes/no item, which did not capture the type, chronicity, or timing of the trauma, nor the mental health effects (if any) of the trauma. Similarly, alcohol use was measured with a three-item screening with relatively high sensitivity that may have produced false positive cases of problematic use. Keeping feasibility and participant burden, and privacy in balance, future research should consider more in-depth stressor assessment. This study also did not fully address cultural norms or religion, which could impact parenting quality and responses to stress, and should be explored further. Possible differences in these processes across cultural, racial/ethnic, or socioeconomic groups also warrant further investigation. Finally, findings may also have been influenced by selection bias, as not all eligible parents completed the survey or interview. Parents who completed this study may have been more interested in parenting support programs.

Despite its limitations, this study demonstrated several strengths, including a focus on perspectives and lived experiences of a racially and socioeconomically diverse group of parents dealing with multiple major stressors. A community-engaged approach that gathers parent perspectives on program delivery and content is likely to result in better uptake of the intervention [47,48,49], which may enhance equity in healthcare access. Another strength included the mixed methods approach, which allows for triangulation of qualitative and quantitative data. Lastly, the focus on addressing parent stress, self-regulation, and emotional skills within the context of positive parenting, with targeted outcomes integrating across social–emotional and lifestyle behavior health, is a novel and innovative approach to improving the health and well-being of families affected by adversity.

### 4.2. Clinical Implications

This study has several implications for healthcare service delivery and practitioners working with children and families affected by adversity. First, given parents’ high interest in addressing their own stress and coping within the context of a parenting support program, practitioners should assess parental stressors and psychological functioning when families initiate child- and family-focused services. For parents who experience high stress, practitioners may consider incorporating stress reduction strategies for parents themselves. For example, several cognitive and behavioral strategies may be effective for parents, such as relaxation techniques, mindfulness practices, coping thoughts, and problem-solving skills. These strategies can be incorporated within child-focused mental healthcare service delivery, as parental mental health is traditionally not addressed in this context. Additionally, considering the high prevalence and reported effects of trauma, practitioners should adopt a strengths-based, trauma-informed approach, which can include providing parents with autonomy and decision-making power (e.g., allowing some flexibility in session material) and creating space for parents to explore the effects of their difficult past experiences on their well-being and parenting. A trauma-informed approach can be adopted across multiple levels of healthcare service delivery and may serve to promote equitable access to services and outcomes for families experiencing racial and economic marginalization. Parents with financial strain, single parenthood, physical illness, mental health concerns, and other related stressors may benefit from connection to case management services, community resources, and other supportive services, as well as enhancing parents’ social support systems through peer support groups or other social connections. Parents in this study expressed interest in programs that simultaneously address children’s social–emotional and lifestyle health; practitioners may consider ways to tailor services to incorporate an emphasis on both aspects of child well-being. Importantly, a flexible approach that is aligned with the needs, strengths, and values of each family is needed to optimally promote resilience and equity among families affected by adversity.

## 5. Conclusions

This study collected formative data that will be used to guide the implementation and evaluation of a parent-based prevention program seeking to address two critical gaps: (1) a lack of sufficient attention on parental stress reduction and self-regulation in existing parent-based programs, particularly for parents experiencing major stressors, and (2) a siloed approach in which social–emotional are lifestyle behavior health are addressed separately. The integration of qualitative and quantitative findings in this study indicate that families are indeed affected by stress across multiple domains; that families under stress still exhibit resilience factors that can be built upon to further support their thriving in the context of adversity; and that parents are interested in programs that integrate across positive parenting strategies, parental stress reduction and coping, and child physical and social–emotional health outcomes.

## Figures and Tables

**Table 1:** Summary of child and caregiver demographic variables.

	Quantitative Subsample (n = 46)	Qualitative Subsample (n = 25)
	M (SD) or N (%)	M (SD) or N (%)
**Child demographic characteristics**		
Age (years)	5.8 (1.78)	5.68 (1.52)
Gender		
Male	24 (52.2)	16 (64)
Female	22 (47.8)	9 (36)
Non-binary/transgender	0 (0)	0 (0)
Race ^1^		
White	22 (47.8)	13 (52)
Black or African American	26 (56.5)	14 (56)
Asian	3 (6.5)	1 (4)
American Indian or Alaskan native	3 (6.5)	2 (8)
Native Hawaiian/other Pacific Islander	1 (2.2)	1 (4)
Middle Eastern or North African	0 (0)	0 (0)
Prefer to describe	1 (2.2)	0 (0)
Ethnicity		
Hispanic	3 (6.7)	2 (8)
**Caregiver demographic characteristics**		
Age (years)	33.75 (5.9)	34.12 (4.97)
Race		
White	22 (46.8)	12 (48)
Black or African American	23 (48.9)	13 (52)
Asian	2 (4.3)	0 (0)
American Indian or Alaskan native	2 (4.3)	0 (0)
Native Hawaiian/other Pacific Islander	0 (0)	0 (0)
Middle Eastern or North African	0 (0)	0 (0)
Prefer to describe	1 (2.1)	1 (4)
Ethnicity		
Hispanic	2 (4.3)	2 (8)
Education		
Some high school	2 (4.3)	0 (0)
High school diploma/GED	6 (12.8)	4 (16)
Some college	19 (40.4)	8 (32)
Trade/technical/vocational training	2 (4.3)	1 (4)
Associate’s degree	5 (10.6)	2 (8)
Bachelor’s degree	7 (14.9)	6 (24)
Master’s degree	4 (8.5)	3 (12)
Professional degree	1 (2.1)	0 (0)
Doctorate degree	1 (2.1)	1 (4)
Relationship Status		
Never married, not in a relationship now	13 (27.7)	7 (28)
Never married, but currently in a relationship	9 (19.1)	5 (20)
Currently married	18 (38.3)	11 (44)
Currently separated or divorced	7 (14.9)	2 (8)
Employment ^2^		
Full-time	19 (40.4)	9 (36)
Part-time	9 (19.1)	6 (24)
No regular employment	6 (12.8)	3 (12)
Homemaker/stay-at-home parent	13 (27.7)	7 (28)
Student	4 (8.5)	1 (4)
Disabled	2 (4.3)	1 (4)
Monthly household income		
Under USD 1000	12 (25.5)	6 (24)
USD 1000–2000	13 (27.7)	9 (36)
USD 2000–3000	6 (12.8)	1 (4)
USD 3000–4000	4 (8.5)	3 (12)
USD 4000–5000	3 (6.4)	1 (4)
USD 5000–10,000	4 (8.5)	2 (8)
Over USD 10,000	5 (10.6)	3 (12)
Number of children aged 3 to 9 years old		
One	23 (48.9)	10 (0.40)
Two	18 (38.3)	12 (0.48)
Three	6 (12.8)	3 (0.12)
Relationship to child		
Mother	45 (97.8)	25 (100)
Grandmother	1 (2.2)	0 (0)

Note. Age and household income variables are summarized by mean (SD). All other demographic variables are summarized by count (percent). ^1^ Some participants selected more than one response for their child’s race. ^2^ Some participants selected more than one response for their type of employment.

**Table 2:** Bivariate correlations among all study variables.

	1	2	3	4	5	6	7	8	9	10	11	12	13	14	15
1. SDQ-TD	-														
2. SDQ-Intern	**0.83 ^ˠ^**	-													
3. SDQ-Extern	**0.72 ^ˠ^**	0.20	-												
4. SDQ-Pro	**−0.56 ^ˠ^**	**−0.39 ^ᶲ^**	**−0.49 ^ˠ^**	-											
5. MAPS-NP	** 0.41 ^ᶲ^**	0.18	** 0.49 ^ˠ^**	−0.19	-										
6. MAPS-PP	−0.31 *	−0.27	−0.20	** 0.53 ^ˠ^**	0.03	-									
7. Parenting-SR	−0.29	**−0.31 ^ᶲ^**	−0.11	0.28	−0.22	0.32 *	-								
8. PDH-CCB	**0.52 ^ᶲ^**	0.28	**0.55 ^ᶲ^**	−0.29	**0.66 ^ˠ^**	0.01	−0.10	-							
9. DASS-PD	**0.40 ^ᶲ^**	0.31 *	**0.32 ^ᶲ^**	−0.25	0.33 *	−0.32 *	−0.26	0.18	-						
10. DASS-PA	**0.42 ^ᶲ^**	**0.40 ^ᶲ^**	0.23	0.05	0.38 *	−0.14	**−0.38 ^ᶲ^**	0.26	**0.66 ^ˠ^**	-					
11. DASS-PS	**0.54 ^ˠ^**	**0.48 ^ˠ^**	0.34 *	−0.30 *	**0.48 ^ˠ^**	−0.32 *	−0.35 *	0.28	**0.80 ^ˠ^**	**0.67 ^ˠ^**	-				
12. PA-WD	0.06	−0.02	0.12	−0.03	−0.02	0.13	0.01	0.29	−0.16	−0.04	−0.10	-			
13. PA-WEKND	−0.07	−0.03	−0.09	−0.01	0.09	0.14	0.03	−0.02	−0.38 *	−0.20	−0.20	**0.52 ^ˠ^**	-		
14. Screen time	0.07	−0.02	0.14	0.28	0.30 *	0.28	0.01	0.17	0.27	0.24	0.24	0.13	−0.14	-	
15. Sleep	0.33 *	0.32 *	0.19	−0.25	0.01	**−0.45 ^ᶲ^**	−0.21	0.18	**0.41 ^ᶲ^**	**0.32 ^ᶲ^**	0.33 *	−0.13	−0.09	−0.03	-
*M*	16.70	5.96	10.74	6.72	2.11	4.33	63.76	14.48	13.26	9.74	18.57	-	-	-	1.72
*SD*	6.08	4.34	3.50	2.54	0.63	0.59	14.52	7.11	11.29	8.62	9.71	-	-	-	1.38
Range	4–31	0–16	4–19	0–10	1–4.10	2–4.96	12–84	0–28	0–42	0–42	0–42	-	-	-	0–5
α	0.89	0.85	0.84	0.92	0.92	0.92	0.91	0.80	0.93	0.83	0.84	-	-	-	0.65

Note. SDQ = Strengths and Difficulties Questionnaire; SDQ-TD = total difficulties score; SDQ-Intern = child internalizing behavior; SDQ-Extern = child externalizing behavior; SDQ-Pro = child prosocial behavior; MAPS = Multidimensional Assessment of Parents; MAPS NP = negative parenting; MAPS PP = positive parenting. Parenting SR = Parenting Self-Regulation Scale. PDH-CCB = Parenting Daily Hassles–Child Challenging Behavior; DASS = depression, anxiety, and stress scale; DASS-PD = parent depression; DASS-PA = parent anxiety; and DASS-PS = parent stress; PA = physical activity; PA WD = PA throughout the weekday inside and outside of school; PA WEKND = PA on the weekend only; screen time = screen time on weekdays and weekends; Sleep = total sleep problems. Statistically significant correlations are bolded. ^ˠ^ *p* < 0.001, ^ᶲ^ *p* < 0.01, * *p* < 0.05.

**Table 3:** Qualitative themes and representative quotes.

Theme	Representative Quote
Theme 1: Family Strengths	*We’re a tightknit family. We do sit around and communicate with our kids a lot. We try to let them be themselves and be children, so we pride ourselves in that. We try to allow them to express themselves as much as possible*. [parent of a 9-year-old; ID 43]
Theme 2: Child Strengths and Challenges	*He is…the most loving and emotional child you would ever come across, [which can be] good and bad. When he feels, he feels. It’s either going to be all love or all the ‘I’m mad’ or all the ‘Oh god, you hurt my feelings.’* [parent of a 6-year-old; ID 7]
Theme 3: Parent Stressors	*I just think a lot of that comes from being a single parent, the stressors of the finances, which also stresses me, the emotional—carrying the weight of both parents, which also, depending how quickly you can resolve certain things, our economy is not exactly built for a single mom of however many. I don’t care who you are, don’t care what your support system is, especially when it’s lacking. There is not much emotional support, community or resources to help … We’re not homeless. We’re not at that point, but it wouldn’t take much. And I don’t think there’s a lot of support or community for that when you’re not at rock bottom.* [parent of a 4-year-old; ID 12]
Theme 4: Effects of Stress on Families	
4a. Impact of Stress on Child Well-being	*If I’m not regulated myself then nobody in this house is regulated.* [parent of a 5-year-old; ID 26]
4b. Impact of Stress on Parenting and Parent–Child Interactions	*When I’m feeling down and having sort of like bad days, it’s I’m much less likely to want to like, go play or just engaged in conversation. I’m more just sort of like, just around. I’m just like a body. So, I’m an adult in the room supervising, but I’m just not super connected and engaged.* [parent of a 6-year-old; ID 3]
4c. Impact of Stress on Parent Well-being	*[Stress] definitely interferes with my sleep, because I worry about it. At times, I’m a heavy drinker, but I would prefer not to drink any alcohol. And so I find myself coping with alcohol or food at times. I think it also causes additional struggle with my ex, because sometimes we don’t agree on how to resolve or work on [Child]’s issues.* [parent of a 7-year-old; ID 56]
Theme 5: Parent Strengths and Strategies to Manage Stress	*Kind of just talking through any issues, either with my husband or family or friends, you know, depending on what it is, so just communicating with different people and getting their advice, their help* [parent of an 8-year-old; ID 4]
Theme 6: Helping Children Deal with Stress	*I will talk to her about something that might be bothering her and really try to attend to her emotions and make sure that she understands her emotions to the best of her ability* [parent of a 3-year-old; ID 16]
Theme 7: Parenting Support Program Opinions and Preferences	*I think stress management and coping techniques for the parents themselves and topics about helping your child with really big feelings…would be really helpful. Maybe even parents need help learning how to name their feelings.* [parent of a 6-year-old; ID 3]

## Data Availability

The data presented in this study are available on request from the corresponding author due to privacy reasons.

## Supplementary Materials

The following supporting information can be downloaded at https://www.mdpi.com/article/10.3390/healthcare13182366/s1. The supporting information includes Table S1: Sample items for screening and survey measures and Table S2: Screening questions.
