# Supplementary material for: Perceptions of Stress, Well-Being, and Intervention Preferences Among Parents Affected by Major Stressors
Source: Healthcare (Basel). 2025 Sep 20;13(18):2366. doi: 10.3390/healthcare13182366 (PMC12469705; doi:10.3390/healthcare13182366)
Supplement: Supplementary file 1 [file healthcare-13-02366-s001.zip › healthcare-3716408-supplementary.pdf]

Table S1. Sample items for screening and survey measures

Construct	Measure	Sample Item
Parent mental health	Patient Health Questionnaire (PHQ-4)	Feeling nervous, anxious, or on edge
Racial Discrimination	Everyday Discrimination Scale	People act as if they think you are not smart
Alcohol use	Alcohol Use Disorder Identification Test-Concise (AUDIT-C)	How often do you have a drink containing alcohol?
Drug Use	Drug Abuse Screen Test (DAST)	Do you use more than one drug at a time?
Child social-emotional functioning	Strengths and Difficulties Questionnaire (SDQ)	Shares readily with other children, for example toys, treats, pencils
Child physical activity	Parent report of physical activity	On a typical weekday, how many minutes does [child] spend outside or in a gym for recess or unstructured free play during school/daycare?
Child sleep	BEARS screening tool	Does [child] have any problems at bedtime?
Parenting practices	Multidimensional Assessment of Parenting Scale (MAPS)	I express affection by hugging, kissing, and holding my child.
Parenting stress	Parenting Daily Hassles Scale (PDH)	The kids won't listen or do what they are asked without being nagged
Parental self-regulation	Parenting Self Regulation Scale (PSRS)	I know what behaviors and skills I want to encourage in my child
Parent psychological well-being	Depression, Anxiety, and Stress Scale (DASS-21)	I couldn't seem to experience any positive feeling at all

Table S2. Screening Questions.

Screening Questions
Are you 18 years of age or older?
Are you the parent or guardian of a child between the ages of 3 and 9 years old?
Are you a primary caregiver for this child (meaning the child lives with you for over half the time)?
Do you have any concerns about your child in the following areas? If you have more than one child between age 3 and 9, think about the child whose behavior, mood, or health concerns you the most. Please select all that apply.
What is your current MONTHLY household income from all sources (BEFORE taxes)? This includes your income from wages/salary earned at a job, your income from other sources, such as unemployment compensation, workers' compensation, SSI, public assistance, child support, and other sources, plus the income of others who work and live in your household. Enter just a number. Do not use commas or decimals. For example, "4000".
How many people live in your household?
What is your gender?
Are you Hispanic or of Latino origin?
Which best describes your race? (Select all that apply).

Note. Screening tools from validated measures not included.
